# BMI and recommended levels of physical activity in school children

**DOI:** 10.1186/s12889-017-4492-4

**Published:** 2017-06-24

**Authors:** Phillipp Schwarzfischer, Martina Weber, Dariusz Gruszfeld, Piotr Socha, Veronica Luque, Joaquin Escribano, Annick Xhonneux, Elvira Verduci, Benedetta Mariani, Berthold Koletzko, Veit Grote

**Affiliations:** 1 Division of Metabolic and Nutritional Medicine, Dr. von Hauner Children’s Hospital, University of Munich Medical Centre, 80337 Munich, Germany; 2 Children’s Memorial Health Institute, Neonatal Intensive Care Unit, 04-736 Warsaw, Poland; 3 Paediatrics Research Unit, Universitat Rovira i Virgili, 43201 Reus, Spain; 4 CHC St. Vincent, -Rocourt, 4000 Liège, Belgium; 5 Deparment of Peadiatrics, San Paolo Hospital, University of Milan, 20142 Milan, Italy

**Keywords:** Physical activity guidelines, Obesity, Sensewear armband, Accelerometer

## Abstract

**Background:**

Physical activity (PA) and its health benefits are a continuous point of discussion. Recommendations for children’s daily PA vary between guidelines. To better define the amount of PA necessary to prevent overweight and obesity in children, further research is needed. The present study investigates children’s compliance to physical activity guidelines (PAGs) and the association between objectively measured PA and body mass index (BMI).

**Methods:**

Participating children were 11 years old (*n* = 419) and part of the European CHOP trial, which was conducted in Germany, Belgium, Poland, Spain, Italy. At least 2 days of PA measurements were collected from each child using a SenseWear™ armband. BMI was calculated from children’s height and weight. Thresholds of min·day^−1^ in PA needed to differentiate between normal and excess weight (overweight/obesity) were determined with Receiver Operator Characteristics (ROC) analysis. Additionally, adjusted linear and logistic regressions models were calculated for group differences and effects of a 5, 15 and 60 min·day^−1^ increases in PA on BMI.

**Results:**

Median time spent in total PA was 462 min·day^−1^ (25th percentile; 75th percentile: 389; 534) and 75 min·day^−1^ (41; 115) in moderate to vigorous PA (MVPA). Girls spent 36 min·day^−1^ less in MVPA than boys and overweight/obese children 24 min·day^−1^ less than normal weight children (linear regression, *p* < 0.001). 63.2% of the children met PAGs of 60 min·day^−1^ in MVPA. The optimal threshold for min·day^−1^ in MVPA determined with ROC analysis was 46 min·day^−1^. Comparing 5, 15 and 60 min·day^−1^ increases in PA revealed that an additional 15 min·day^−1^ of vigorous PA had the same effect as 60 min·day^−1^ of MVPA. Sedentary time and light PA showed contrary associations to one another, with light PA being negatively and sedentary time being positively associated with excessive weight.

**Conclusions:**

Current PAGs are met by 2/3 of children and seem appropriate to prevent excess weight in children. An official recommendation of daily 15–20 min of vigorous PA and further reduction of sedentary time could help to fight youth overweight and thus be of potential public health importance.

**Trial registration:**

ClinicalTrials.gov Identifier: NCT00338689. Registered: June 19, 2006 (retrospectively registered).

## Background

Physical activity (PA), as stated by the World Health Organization (WHO) and confirmed by numerous studies, provides numerous health benefits [1], helps to prevent chronic diseases [2], balance daily energy expenditure (EE) and maintain a healthy body composition [3]. In 2008 the European Commission presented Physical Activity Guidelines (PAGs) for the European Union [4]. They cite the recommendations of the WHO: pre-school children should accumulate a minimum of 180 min·day^−1^of PA, children and adolescents (4–17 years) at least 60 min·day^−1^ in moderate- to vigorous-intensity PA (MVPA) and for adults a minimum of 30 min·day^−1^ in MVPA should be achieved [5]. While these recommendations specify the amount of MVPA for children, information about recommended daily time in other intensity levels of PA is lacking. The WHO recommends vigorous-intensity activities at least 3 times per week [5], but does not state an appropriate amount in min·day^−1^. Additionally, sedentary behaviour has changed dramatically in recent years [6] and is not specifically mentioned in current guidelines. Screen time of children and adolescents increased [7] and evidence of a causal relationship between sedentary behaviour and all-cause mortality continues to grow [8]. Large population data published in 2012 showed that, worryingly, 81% of the 13–15 year-old children worldwide are too inactive and do not meet current PAGs [9].

Overweight and obesity in children is still on the rise [10] and excessive weight in younger years negatively effects later health [11, 12]. Increasing PA, especially accompanied by healthy eating, can tackle this growing public health problem [13]. However, studies reported various results, when looking at activity and its effect on BMI. A review by Jansen and Leblanc [1] found only weak to modest relationships between PA and overweight/obesity in school-aged children. Mitchell et al. [14], confirmed these modest effects in normal weight children, but saw stronger associations in overweight children and demonstrated that PA is a valuable tool in fighting obesity.

With the rise and improvement of accelerometers in the last decade a re-evaluation of current PAGs is necessary. In light of this advancement, recent studies examined the association between LPA and weight in children, but found contrary results. For instance, studies by Kwon et al. [15] and Treuth et al. [16] found an negative correlation between LPA, body composition and BMI, whereas other studies reported no association between LPA and weight status [17, 18].

An ongoing examination of PA and sedentary time and its relation to BMI is essential to improve current PAGs [19] and help define the PA levels necessary to prevent adverse weight status in young people. The aim of this study is to examine objectively measured PA of children and their compliance to PAGs of 60 min·day^−1^ in MVPA per day and investigate whether these guidelines are adequate. Additionally, we analyse different intensities and durations of PA and sedentary time and how they are associated with BMI.

## Methods

### Study subjects and design

The underlying sample of children is part of the Childhood Obesity Project (CHOP) cohort. This European project was initiated in 2002 and recruited 1678 infants in Germany, Belgium, Italy, Spain and Poland during their first 8 weeks of life. The initial aim of the study was to investigate if different levels of dairy protein intake in early life influence BMI and body composition in later life [20]. Data for this analysis was collected during the 11 years follow up and represents a cross-sectional sample of 589 children, of which 445 had complete data on PA and anthropometry. Data collection was coordinated by 5 study teams in 8 urban and sub-urban areas: Germany (Nuremberg, Munich), Italy (Milano), Belgium (Brussels, Liege), Poland (Warsaw) and Spain (Reus, Tarragona). The trial was approved by ethics committees in each study centre and written informed consent was obtained from parents and children. All research was performed in accordance with the Declaration of Helsinki.

### Activity assessment

Physical activity was measured using the SenseWear™ Armband 2 (SWA) (Body Media Inc., Pittsburgh, PA). The device is worn over the right triceps brachii muscle and incorporates five sensors: two-axis accelerometer (for movement patterns and step-count), galvanic skin response, skin temperature, near body temperature sensor and heat flux [21]. According to the study protocol, children were told to wear the SWA on 3 consecutive days for at least 20 h·day^−1^. This time frame was proposed by Trost et al. [22] for accelerometer studies and applied in other studies where SWA measurements were taken [23, 24]. Collected data was exported via the Professional InnerView Software 6.1 (Body Media Inc., Pittsburgh, PA). This software calculates the EE from the sensor parameters together with anthropometric data (gender, age, height, weight, BMI, handedness, smoking status). Physical activity intensity is classified by Metabolic Equivalents of Task (MET). METs are a practicable unit, defined by Jette et al. [25], which are commonly used to classify activities based on their EE. Studies have shown that the SWA is a valid tool for measuring EE and PA in children and adolescents [23, 24].

PA was categorized into 4 groups based on recommendations by Trost et al. [26]: light PA (LPA; 1.5–3.9 METs) includes standing, light walking, stretching, washing dishes; moderate PA (MPA; 4–5.9 METs) includes brisk walking, stair-climbing, water aerobics, biking on level ground; vigorous PA (VPA; > = 6 METs) encompasses mostly exercise activities like jogging or soccer. Time spent in activity below 1.5 METs minus time spent lying and sleeping, as recorded by the SWA, was considered sedentary behaviour.

### Anthropometric measurement

Anthropometric measurements were conducted during the 11 year follow-up visit at all study sites. Standard operation procedures relied on the WHO’s Growth Reference Study [27]. Study personnel at each site were trained and the same weight scale (SECA™ 702) and stadiometer (SECA™ 242) were used to measure weight and height at each site. Body mass index (BMI) was calculated (weight [kg]·height [m]^−2^) and transformed into BMI z-scores, adjusted for gender and age according to the WHO growth standards [27]. Cut-off values for weight status were as follows: normal weight BMI z-score > = −2 SD and <= +1SD, overweight BMI z-score > +1 SD and obese BMI z-score > +2 SD [27, 28]. Children with a BMI z-score < −2 SD were excluded as underweight children were not the focus of this analysis and could bias results.

### Covariates

Study country, season of measurement and gender of child were regarded as fixed covariates for adjustment in regression models. Additional data from parents was collected during the initial CHOP study visit (within 8 weeks after child’s birth) and were also considered: educational status and nationality of parents (one or both parents not from study country), age of mother at birth and BMI of mother before pregnancy.

#### Data analyses

Data is reported as mean (μ) ± standard deviation (SD) for continuous variables and as number (n) and percentage (%) for factors. PA parameters were skewed and reported as median (25th percentile; 75th percentile). Student’s t-test and one-way ANOVA were used for group differences in parametric variables and Kruskal-Wallis one-way ANOVA was used for skewed variables. Linear regression models including covariates (as mentioned above) with PA, MVPA and sedentary time as dependent variables were calculated for adjusted group differences. Compliance to PAGs is reported as number (n) and percentage (%) of children meeting recommendations. Additionally, odds ratios (OR) for adherence to PAGs (yes/no) were calculated with adjusted logistic regression models.

To define amount of PA needed to prevent excess weight in children, Receiver Operating Characteristic (ROC) curve analysis was used. ROC analysis can provide optimal cut-off values for time spent in specific levels of PA to differentiate between being overweight/obese and normal weight. Sensitivity (correctly identify an overweight or obese child) and specificity (correctly identify a normal weight child) for every possible cut-off was calculated and optimal thresholds were determined with the Youden-index as the maximum value of [*J*]:

$$ J=\mathrm{Sensitivity}+\mathrm{Specificity}\hbox{--} 1 $$

Area under the curve (AUC) ranges from 0.5 (test variable predicts outcome only by chance) and 1.0 (perfect prediction) and reflects the quality of the test variable in predicting the outcome.

The effects of an increase (5, 15 and 60 min) in different intensity levels of PA on weight status (normal or overweight/obese children; based on BMI z-score) were calculated, using logistic regression models adjusted for covariates. All regression models were controlled for normal distributed residuals. *P*-values ≤0.05 were seen as significant.

After export from the SWA, PA data was processed using R 3.3.0 (The R Foundation for Statistical Computing). All statistical analysis was performed using ‘IBM SPSS Statistics for Windows’ version 23 (IBM Corp., Armonk, N.Y., USA).

## Results

Complete data about activity, anthropometry and covariates was collected from 445 children. Twenty-six children had to be excluded due to less than 2 days of recording data, underweight or technical reasons. There were no differences (*p* > 0.05) in PA or anthropometric variables between children with 2 days of recording and children with 3 or more days. For analysis children with 2 or more days and anthropometric data were included (*n* = 419).

Study characteristics are shown in Table 1. A total of 134 (32.0%) of the children were classified as overweight/obese. BMI at 11 years of age was significantly different between countries (BMI z-scores; *p* = 0.009), ranging from Germany with lowest mean BMI (μ: 17.89 ± SD: 3.07; BMI-z: 0.08 ± 1.15) to Italy with highest mean BMI (19.44 ± 3.27; BMI-z: 1.18 ± 0.69); no gender differences were seen. However, girls (148.6 cm ± 7.2) were significantly taller by 1.5 cm (*p* = 0.026) than boys (147.1 cm ± 6.5).

**Table 1:** Anthropometric data/country of participating 11 year olds and numbers of physical activity measurements per season

	μ (SD)
Weight (kg)	41.5 (9.4)
Height (cm)	147.9 (6.9)
BMI	18.8 (3.3)
BMI z-score	0.5 (1.2)
	*n* (%)
*N* Total	419 (100%)
Gender	
Male	190 (45.3%)
Female	229 (54.7%)
BMI	
Normal	285 (68.0%)
Overweight/Obese	134 (32.0%)
Country	
Germany	62 (14.8%)
Belgium	60 (14.3%)
Italy	103 (24.6%)
Poland	62 (14.8%)
Spain	132 (31.5%)
Season	
Winter	137 (32.7%)
Spring	105 (25.1%)
Summer	65 (15.5%)
Autumn	112 (26.7%)

### Adherence to guidelines and differences in PA

Table 2 displays the distribution of time spent in different levels of PA and sedentary behaviour. Differences in amount of min·day^−1^ in PA based on weight status (*p* < 0.001), country (*p* = 0.034) and season (*p* = 0.005) were observed. Children with excess weight spent more time in sedentary behaviour, Median = 433 min·day^−1^ (25th percentile; 75th percentile: 362; 504) and less time in physical activity, 426 min·day^−1^ (355; 496) than normal weight children (sedentary: 374 min·day^−1^ (330; 446), *p* < 0.001; PA: 478 min·day^−1^ (408; 541), *p* < 0.001). Gender difference were visible, with boys spending more time at higher intensities (MPA: *p* < 0.001, VPA: *p* < 0.001 and MVPA: *p* < 0.001) and girls more time at lower intensities (LPA: *p* < 0.001). PAGs of 60 min·day^−1^ were met by 63.2% of the children. Boys had a higher compliance (80.0%) than girls (50.0%).

Adjusted estimates of the linear regression model for time spent in PA revealed that overweight children spent 47 min·day^−1^ (95% CI: -69, −25; *p* < 0.001) less in PA than children of a normal weight; children in Italy (estimate: −36 min·day^−1^; 95% CI: -65, −8; *p* = 0.013) or Poland (estimate: −37 min·day^−1^, 95% CI: -69, −4; *p* = 0.028) spent less time daily in PA than children from Spain. Overall time in PA during winter was 39 min·day^−1^ (95%CI: -69, −9; *p* = 0.012) lower compared to summer. Analysis of results also showed that weight status and gender-based differences were observed for MVPA: girls spent 36 min·day^−1^ (95% CI: -46, −25; *p* < 0.001) less in MVPA than boys; overweight/obese 24 min·day^−1^ (95% CI: -36, −13; *p* < 0.001) less than normal weight children. Seasonal differences prevailed, with winter being associated with the lowest time spent on MVPA (estimate: −26 min·day^−1^; 95% CI: -42, −10; *p* = 0.001) compared to summer. No Interaction between season and country were seen.

Concerning adherence to PAGs, girls had 4.67 (95% CI: 2.87, 7.62; *p* < 0.001) times higher odds than boys to fall below current recommendations. Overweight and obese had more than twice the odds (OR 2.60; 95% CI: 1.55, 4.38; *p* < 0.001) than normal weight children.

### Threshold determination

Results from the ROC analyses can be found in Table 3. The overall AUCs for PA variables and sedentary time were significantly (*p* < 0.05) greater than 0.5. With rising intensity levels of PA, the AUC increased and the threshold for min·day^−1^ in PA progressively decreased (from LPA cut-off: 348 min·day^−1^ to VPA cut-off: 5 min·day^−1^). For MVPA, the AUC was 0.62 (95%CI: 0.56, 0.68) and resulted in a threshold of 46 min·day^−1^. When stratifying for gender, girls’ thresholds were comparable with the overall sample results. Boys had higher cut-offs for minutes per day in MPA, VPA and MVPA needed differentiate the weight status.

**Table 3:** Cut-off points for min·day^−1^ in physical activity^a^/sedentary to differentiate between normal weight and overweight/obese^b^ children

	AUC (95%CI)	Youden index	Cut-off (min·day^−1^)	Sensitivity	Specificity
Total					
Sedentary	0.64 (0.58; 0.70)	.255	388	0.69	0.57
LPA	0.58 (0.52; 0.64)	.205	348	0.71	0.50
MPA	0.61 (0.55; 0.67)	.234	38	0.79	0.45
VPA	0.64 (0.58; 0.70)	.260	5	0.79	0.47
MVPA	0.62 (0.56; 0.68)	.230	46	0.78	0.45
Male					
Sedentary	0.63 (0.54; 0.72)	.249	382	0.77	0.48
LPA	0.60 (0.51; 0.68)	.286	344	0.64	0.65
MPA	0.63 (0.54; 0.72)	.239	69	0.62	0.62
VPA	0.67 (0.58; 0.75)	.290	20	0.55	0.74
MVPA	0.65 (0.56; 0.73)	.276	76	0.77	0.51
Female					
Sedentary	0.65 (0.57; 0.72)	.288	410	0.59	0.69
LPA	0.55 (0.47; 0.64)	.137	349	0.78	0.36
MPA	0.63 (0.55; 0.71)	.263	39	0.67	0.59
VPA	0.66 (0.58; 0.74)	.317	6	0.65	0.67
MVPA	0.64 (0.56; 0.72)	.271	46	0.66	0.61

*P*-values indicate AUC significantly > 0.50

^a^LPA: light physical activity (1.5–3.9 METs), MPA: moderate physical activity (MPA; 4–5.9 METs), VPA: vigorous physical (VPA; > = 6 METs)

*MVPA: moderate to vigorous physical activity*

^b^Classification through BMI z-scores, calculated according to WHO standards: normal weight > = − 2 SD and < = + 1SD, overweight/obese > +1 SD

**Table 2:** Minutes·day^−1^ spent physically active^a^/ inactive and number (%) of children who adhered to activity recommendations

	*n*	Sedentary	PA	LPA	MPA	VPA	MVPA	Adherence PAGs^b^
Total	419	391 (333; 473)	462 (389; 534)	379 (318; 429)	60 (35; 93)	11 (4; 24)	75 (41; 115)	265 (63.2%)
Gender								
Male	190	404 (338; 480)	458 (371; 530)	**353 (297; 396)**	**71 (54; 107)**	**19 (9; 33)**	**95 (65; 139)**	152 (80.0%)
Female	229	383 (330; 458)	473 (395; 539)	**399 (346; 447)**	**47 (27; 74)**	**8 (3; 15)**	**59 (33; 92)**	113 (49.3%)
BMI								
Normal	285	**374 (330; 446)**	**478 (408; 541)**	**384 (333; 431)**	**65 (41; 95)**	**13 (6; 26)**	**82 (51; 126)**	198 (69.5%)
Overweight/Obese	134	**433 (362; 504)**	**426 (355; 496)**	**352 (288; 422)**	**47 (27; 74)**	**7 (2; 17)**	**60 (30; 102)**	67 (50.0%)
Country								
Germany	62	**394 (333; 484)**	**452 (390; 536)**	**367 (317; 417)**	**65 (39; 92)**	**16 (8; 26)**	**79 (50; 115)**	44 (71.0%)
Belgium	60	**373 (299; 423)**	**451 (401; 537)**	**387 (329; 453)**	**55 (32; 84)**	**12 (4; 22)**	**69 (40; 106)**	36 (60.0%)
Italy	103	**428 (350; 503)**	**452 (367; 524)**	**381 (319; 422)**	**50 (27; 84)**	**9 (2; 20)**	**59 (28; 100)**	51 (49.5%)
Poland	62	**426 (383; 511)**	**452 (362; 518)**	**353 (288; 398)**	**68 (38; 107)**	**10 (5; 21)**	**78 (48; 128)**	43 (69.4%)
Spain	132	**365 (323; 440)**	**491 (411; 540)**	**391 (337; 437)**	**67 (40; 95)**	**12 (5; 28)**	**82 (47; 125)**	91 (68.9%)
Season								
Winter	137	**409 (350; 492)**	**439 (364; 515)**	369 (307; 418)	**55 (31; 80)**	10 (3; 20)	**65 (36; 106)**	79 (57.7%)
Spring	105	**407 (337; 488)**	**461 (391; 540)**	374 (326; 422)	**61 (30; 92)**	13 (4; 25)	**77 (37; 113)**	64 (61.0%)
Summer	65	**360 (311; 415)**	**496 (419; 555)**	381 (321; 427)	**77 (39; 117)**	14 (6; 25)	**92 (51; 143)**	47 (72.3%)
Autumn	112	**389 (320; 452)**	**480 (414; 540)**	386 (338; 445)	**68 (39; 94)**	12 (6; 26)	**80 (49; 126)**	75 (67.0%)

*Values are Median (25th percentile; 75th percentile) unless otherwise stated*

*Bold: significant group differences (p < 0.05)*

^*a*^ *PA: physical activity, LPA: light physical activity (1.5–3.9 METs), MPA: moderate physical activity (MPA; 4–5.9 METs), VPA: vigorous physical (VPA; > = 6 METs), MVPA: moderate and vigorous physical activity*

^*b*^ *Adherence to physical activity guidelines (PAGs): Number of children adhering to current physical activity guidelines of 60 min·day* ^*−1*^ *in MVPA (percentage of group in column)*

### Associations of different levels of PA with BMI

**Fig. 1:**
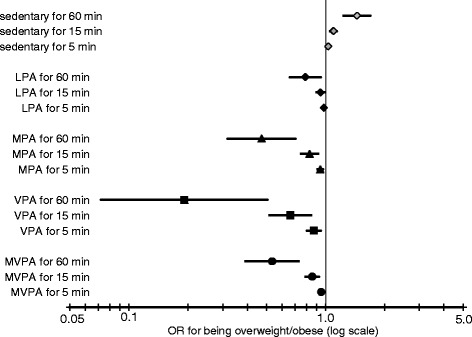
Odds Ratios (OR) and 95% confidence intervals of being overweight and/or obese for an increase in different intensities of physical activity, calculated with logistic regression models, adjusted for gender, country, season, education and nationality of parents, age and BMI of mother at birth. Note: ‘min’ stand for 5/15/60 min·day^−1^ increase in PA and sedentary time, with each line representing a separate model. LPA: light physical activity (1.5–3.9 METs), MPA: moderate physical activity (4–5.9 METs), VPA: vigorous physical (> = 6 METs), MVPA: moderate to vigorous physical activity

Figure 1 shows the odds ratios of being overweight/obese with an increase of 5, 15 and 60 min·day^−1^ in different PA intensities. 60 min·day^−1^ of LPA (OR: 0.79; 95% CI: 0.66, 0.95; *p* < 0.001) had about the same effect as 15 min·day^−1^ of MPA (OR: 0.83; 95% CI: 0.75, 0.96; *p* < 0.001) or MVPA (OR: 0.86; 95% CI: 0.79, 0.92; *p* < 0.001) and just 5 min·day^−1^ of VPA (OR: 0.87; 95% CI: 0.80, 0.95; *p* = 0.001). An increase of MVPA (OR: 0.53; 95% CI: 0.39, 0.73; *p* < 0.001) by an hour had the same effect as an hour of MPA (OR: 0.47; 95% CI: 0.32, 0.70; *p* < 0.001) or a 15 min·day^−1^ increase of VPA (OR: 0.66; 95% CI: 0.52, 0.84; *p* = 0.001. Testing for interaction between gender and levels of PA showed no effects.

Analysis was repeated with children, who had at least 3 days of recording (*n* = 353), but showed no major differences. ROC analysis cut-offs remained similar, with two exceptions for the cut-off of boys’ sedentary time (471 min·day^−1^; difference: +89 min·day^−1^) and VPA time (5 min·day^−1^; difference: −15 min·day^−1^). Results of logistic regression models, examining the association between different duration and intensities of PA and sedentary time and weight status, were similar and remained significant.

## Discussion

Results of this study suggest that PA is associated with BMI and current PAGs can be supported, as 60 min·day^−1^ of MVPA showed a meaningful risk reduction of excess weight gain. Approximately two thirds (63.2%) of the 11-year-old children in this study met WHO PAGs. Time spent in PA differed by gender (boys were more active than girls) and weight status (normal weight children were more active than overweight and obese children). Country differences were present, but showed no apparent regional patterns. An additional increase in time spent in higher intensity levels of PA seems to be beneficial for overweight prevention, as an additional 5 min·day^−1^ of VPA showed the same association with weight status, as an additional 15 min·day^−1^ of MPA. LPA was associated with a lower weight status, while sedentary behaviour was associated with excess weight.

### Adherence to guidelines and differences in PA

Reported prevalence of children meeting the 60 min·day^−1^ of MVPA in previous studies ranges from 80% non-adherence [9] to 80% adherence [29]. These variations can be the result of a number of causes. As our and other studies have shown, is that PA and adherence to PAGs varies by country or region. For example, an observational study within the ENERGY-project examining PA of children from 5 different European countries, found rather large country differences [30]. One solution to tackle these differences could be the implementation of national or regional guidelines [5]. Recent review by Kahlmeier et al. [31] however, identified a lack of national PAGs in most European countries.

In addition to regional differences, the differences in adherence and volume of PA by gender [30, 32, 33] and weight status [24, 34] can been seen in several international studies. Despite growing evidence of these discrepancies, PAGs fail to take these differences into account, as causal mechanisms are not completely understood. One explanation could be the influence of friendship and peers on physical activity. This was seen in a qualitative study by Carlin et al. [35], with male peer groups encouraging each other to be active, while in girls’ and overweight/obese groups, participation in sports had mostly negative connotations. Carlin et al. [35] emphasized that interventions and guidelines need to be tailored not just to gender, but also to groups with low PA levels, like overweight or obese children.

Another reason for the wide range of reported prevalence of children meeting PAGs, could be the lack of common cut-offs for MVPA. Current PAGs were established in the last decade and are mostly based on self-reported estimates of PA. However, subjective measurement tends to overestimate PA when compared to accelerometry [36]. The technological development in the last 10 years has led to better opportunities to objectively quantify PA in larger populations. However, with new technologies it is important to validate tools independently and find a clear definition for cut-offs between intensity levels of PA. The cut-offs in this study where based on EE and categories proposed by Trost et al. [26], to make results comparable to other samples. However, amount and intensity of PA can be measured in many different ways: e.g. steps [37], which are easy to understand and used in everyday life, or more complex definitions through fuzzy logic [38]. To date there is no consensus on what defines light, moderate or vigorous PA. Thus, 60 min·day^−1^ of MVPA recommended by PAGs, can be broadly interpreted.

### Threshold determination

ROC analyses thresholds in the overall sample were comparable to current PAGs. Other multinational studies examining cut-offs for objectively measured PA found similar results, though thresholds tend to be higher. The worldwide sample of the ISCOLE study identified an optimal threshold of 55 min·day^−1^, with a higher cut-off for boys (65 min·day^−1^) than girls (49 min·day^−1^) [39]. These gender differences were also visible in the European HELENA study, where ROC analysis was conducted in 2094 adolescents (threshold MVPA overall: 71 min·day^−1^; boys: 56 min·day^−1^; girls: 51 min·day^−1^) [40]. Recent results reported by Laguna et al. [41], analysing recommended levels of PA in 439 Spanish children aged 8–10 years from the European Youth Heart Study, found thresholds of 79 min·day^−1^ of MVPA and 50 min·day^−1^ in VPA. Different thresholds can be mostly traced back to different measurement devices and methods, as well as different populations. A problem of our and similar studies is, that AUCs of the ROC analysis were close to 0.5 and the impact and precision of the test can be debated. Nevertheless, with rising intensity levels of PA (from light to vigorous) AUCs and the quality of the results increase. Time spent in VPA together with time spend in sedentary behaviour seem to be the best parameters, discriminating between normal and excess weight. These results are supported by the effects seen in our logistic regression analysis (Fig. 1).

### Associations of different levels of PA with BMI

International PAGs state that in addition to 60 min·day^−1^ of MVPA, children and adolescents should engage in VPA at least 3 days per week, but no recommended duration is given. The importance of higher levels of PA has been seen in other cross-sectional or interventional studies about PA and obesity. For instance, Ness et al. [42] suggested, based on a cross-sectional analysis of 5500 12-year-old children as part of the Avon Longitudinal Study of Parents and Children (ALSPAC), that higher intensity PA may be more important than total activity. Laguna et al. [41] supported this statement and emphasized the importance of VPA in preventing overweight and obesity. An interventional study by de Araujo et al. [43] showed that high intensity training can be a proficient method for improving health parameters in obese children. In our study an increase of just 15–20 min·day^−1^ of VPA was comparable to an increase of 60 min·day^−1^ of MVPA in reducing the risk of excess weight. This indicates that an appropriate amount for a VPA recommendation could be 15–20 min·day^−1^, this however needs confirmation from other studies.

Recommendations based on the effectiveness of a certain amount of MVPA or VPA should be treated with caution. They may lead to the conclusion that when children spend 60 min·day^−1^ in MVPA or 20 min·day^−1^ in VPA, it does not matter how much time they spent in sedentary time for the rest of the day. Chastin et al. [44] showed that there are complex effects to be considered when trying to replace one behaviour by another (e.g. sedentary time with LPA). Additionally, it is not always feasible to convince children with an inactive lifestyle, to do VPA right away; to start at lower levels PA might be more efficient. The majority of current PAGs agree with the recommendation of 60 min·day^−1^ of MVPA for children. However, it remains unclear how to spend the other 23 h of the day. The Canadian 24-Hour Movement Guidelines were the first to offer an holistic approach to recommendations for sleeping time, PA and sedentary behaviour [45]. PA can be spilt into two parts: LPA and MVPA. LPA is seen as baseline activity according to United States Department of Health and Human Services [46] and MVPA as health-enhancing PA on top of that baseline. Together with sedentary time, LPA is the main component of activity during waking hours, but showed minimal effects on BMI in our study. Despite minor effects on BMI, Pate and al. [6] emphasized the importance to differentiate between LPA and sedentary time. Studies in adults and children have shown that LPA can be a good basis to reduce the negative health effects of sedentary behaviour [15, 47]. Results of our study confirm that LPA reduces the risk of being overweight, while sedentary time is more likely to increase the risk. Future interventions and guidelines should aim to keep sedentary time to a minimum and whenever possible promote LPA. Light intensity activities, like walking to school or playing active videogames can be a more feasible replacement for sedentary time, than MVPA or VPA, as hypothesized by Healy et al. [47].

### Strengths and limitations

One strength of this study is PA measurement using the SWA, since unlike common used accelerometers, it is possible to include weight bearing activities into measurement. Furthermore, the multicentre design with children from 5 European countries and the complete standardisation of the study protocols and measurement tools for every site, gives a broad overview of the PA situation in Europe.

Due to the cross-sectional design of the study, statements about the cause and effect relationships and direction of the association are not possible. Measurement with the SWA has both benefits and downfalls. As with most accelerometers, it cannot be worn in water and cannot effectively measure activities like cycling. The sensewear algorithms seem to improve in measuring children’s EE [48], yet they are prone to under- and overestimate EE in the aforementioned situations. It is possible that more active kids took part in this study and were more likely to accept wearing the SWA, as accelerometer measurement was not mandatory during the CHOP trial. Furthermore, it is unknown, if wearing a sensor changes the PA behaviour of participants and increase their motivation to be more active than usual. Finally, it is only possible to measure PA with accelerometers for a short time frame (few days to week), due to memory and energy limitations of the sensor. Measurements in this study were conducted through the whole year and analysis was adjusted for season, keeping influence of time of measurement to a minimum.

## Conclusions

Results of this study have shown that two thirds of the children met current PAGs and recommendations of 60 min·day^−1^ of MVPA can help prevent excess body weight in children. Despite the fact that children appear to meet current PAGs, overweight and obesity are still on the rise and continue to be a major public health problem. One solution to this could be an additional recommendation of daily 15 min to 20 min in VPA, as this timeframe seem to provide meaningful risk reduction of overweight and obesity. Additional focus should be placed on reducing sedentary time and replacing it with light activity, like walking to school or activity programmes during recess. PA and its promotion can be valuable tools for controlling the body weight of children and subsequently improve their long-term health. Ongoing research in the field of PA is needed to react to changes in lifestyle and monitor activity levels during the early years.

## **Abbreviations**

ANOVA: Analysis of Variance
AUC: Area under the Curve
BMI: Body mass index
CHOP: Childhood obesity project
CI: Confidence Interval
EE: Energy expenditure
LPA: light physical activity
MET: Metabolic Equivalents of Task
min: minutes
MPA: Moderate physical activity
MVPA: Moderate to vigorous physical activity
OR: Odds ratio
PA: Physical activity
PAGs: Physical activity guidelines
SWA: Sensewear armband
VPA: Vigorous physical activity
WHO: World health organisation
